# Enhancing Efficiency of Nitrate Reduction to Ammonia by Fe and Co Nanoparticle-Based Bimetallic Electrocatalyst

**DOI:** 10.3390/ijms25137089

**Published:** 2024-06-28

**Authors:** Irina Kuznetsova, Olga Lebedeva, Dmitry Kultin, Mikhail Mashkin, Konstantin Kalmykov, Leonid Kustov

**Affiliations:** 1 Department of Chemistry, Lomonosov Moscow State University, Moscow 119991, Russia; kuznetsowair@yandex.ru (I.K.); lebedeva@general.chem.msu.ru (O.L.); kbkalmykov@mail.ru (K.K.); lmk@ioc.ac.ru (L.K.); 2 N.D. Zelinsky Institute of Organic Chemistry, Russian Academy of Sciences, Leninsky Prospect 47, Moscow 119991, Russia; 3 Institute of Ecology and Engineering, National Science and Technology University “MISiS”, Leninsky Prospect 4, Moscow 119049, Russia

**Keywords:** synthesis of ammonia, nitrate reduction, electrocatalysis, sustainable chemistry, energy chemistry, Fe/Co catalyst

## Abstract

The green and sustainable electrocatalytic conversion of nitrogen-containing compounds to ammonia is currently in high demand in order to replace the eco-unfriendly Haber–Bosch process. Model catalysts for the nitrate reduction reaction were obtained by electrodeposition of metal Co, Fe, and bimetallic Fe/Co nanoparticles from aqueous solutions onto a graphite substrate. The samples were characterized by the following methods: SEM, XRD, XPS, UV–vis spectroscopy, cyclic (and linear) voltammetry, chronoamperometry, and electrochemical impedance spectroscopy. In addition, the determination of the electrochemically active surface was also performed for all electrocatalysts. The best electrocatalyst was a sample containing Fe-nanoparticles on the layer of Co-nanoparticles, which showed a Faradaic efficiency of 58.2% (E = −0.785 V vs. RHE) at an ammonia yield rate of 14.6 μmol h^−1^ cm^−2^ (at ambient condition). An opinion was expressed to elucidate the mechanism of coordinated electrocatalytic action of a bimetallic electrocatalyst. This work can serve primarily as a starting point for future investigations on electrocatalytic conversion reactions to ammonia using model catalysts of the proposed type.

## 1. Introduction

Liquid hydrogen carriers (LHC), such as liquid organic hydrogen carriers (LOHC) and ammonia (NH_3_), are a promising and environmentally friendly alternative to liquid fuels [1], the usage of which increases the CO_2_ content in the atmosphere. Traditionally, catalytic methods have been used to produce LHC. Recently, however, the attention of researchers has increasingly turned to electrochemical methods. A comparison of these methods [2,3] shows the advantage of the latter, since the synthesis can be carried out at room temperature and hydrogen can be obtained in situ, most often from water.

Promising methods for ammonia synthesis are electrochemical, electrocatalytic, photocatalytic, photoelectrocatalytic, and bio-catalytic [4]. Electrocatalytic methods are the most attractive because they combine the advantages of catalytic and electrochemical methods. The advantages of the latter are the use of an optimum potential or current and the ability to replace known industrial processes [5]. Moreover, the advantage of electrochemical methods of ammonia synthesis over the traditional Haber–Bosch process in terms of greenhouse gas formation was shown [4]. Electrochemical synthesis of ammonia can be carried out not only in aqueous electrolytes but also in ionic liquids and solid electrolytes [6].

Various nitrogen-containing compounds can be used for electrocatalytic production of ammonia. The electroreduction reactions of nitrogen (NRR), nitrogen oxides (NO_x_RR), nitrite (NO_2_RR), and nitrate (NO_3_RR) are actively investigated [7,8,9,10,11,12,13,14,15,16,17]. Two fundamentally different NRR mechanisms are reviewed [8]: dissociative and associative. The first mechanism requires the use of a highly efficient catalyst to break the inert triple bond in the nitrogen molecule. Free energy diagrams for the electrochemical synthesis of ammonia obtained by density-functional theory (DFT) calculations are presented [10]. The limiting stage can be the reductive adsorption of nitrogen to form a *N_2_H particle or the simultaneous transfer of protons and electrons to form a *NH_2_ particle. Based on thermodynamic data [16], the most promising way is the nitrate reduction reaction compared to other substances. The mechanism of NO_3_RR in acidic, neutral, and alkaline media has been described in detail [7,12]. Moreover, NO_3_RR is significant from the point of view of environmental problems as a means of nitrate removal from wastewater [15,16]. The application of NO_3_RR in the water treatment of nitrate has some serious limitations that do not yet allow this process to be implemented on an industrial scale. These are, firstly, the multi-step electron and proton transfer, and secondly, the low concentration of ions to be removed. While producing ammonia by electrocatalytic reduction, a side reaction of hydrogen evolution (HER) is possible, since the reaction potential of ammonia synthesis in an alkaline medium is E° = −0.132 V (vs. SHE, pH = 14) [17]:

NO_3_^−^_(aq)_ +6H_2_O_(l)_ +8e^−^ = NH_3(g)_ + 9OH^−^_(aq)_.

The hydrogen release is also preferable from the kinetic point of view, because fewer electrons are required [8]. Based on the above, one of the criteria for the NO_3_RR selectivity [18] is the evaluation of the NO_3_RR selectivity compared to HER. Other important characteristics of electrocatalytic reduction are the Faradaic efficiency (FE, %), ammonia yield rate (mol s^−1^), turnover frequencies (TOF), energy efficiency (EE, %), and NO_3_^−^ conversion. To determine these characteristics, it is necessary to know the concentration of ammonia in the solution obtained after reduction. The spectrophotometric method, ^1^H NMR spectroscopy, enzymatic and fluorometric methods, ion-selective electrodes, conductivity, and titrimetric methods, ion chromatography, and gas chromatography (GC) are all used for this purpose [9,19,20]. An indophenol spectrophotometric method was compared with GC [19]. The comparison showed excellent correlation between the results. Linear voltammetry (LsV) in a solution containing nitrate ions is used to determine the intermediate stages of reduction and can evaluate the reaction efficiency and catalyst stability [21,22].

Electrode-catalysts for electroreduction reactions should provide high activity, selectivity, and stability [22]. Several reviews [7,20] outline the strategy for the preparation of efficient catalysts, including porous materials, using bimetals and alloys, single-atom catalysts (SAC), and modification by hetero- and nanostructures.

Much attention is paid to finding the relationship between crystal orientation and catalytic properties. For example, the more thermodynamically favorable for NO_3_RR performance are the facets Cu(100) and Cu(111) [23]. Moreover, the competition between HER and NO_3_RR was found to be highly pH-dependent. Through DFT calculations, suitable pH ranges for NO_3_RR on Cu(111), Cu(100), and Cu(110) were found. It was shown through DFT calculations [24] that the (100) facet is more active towards NO_3_RR than the (111) facet, especially at higher pH, due to lower thermodynamic and kinetic barriers.

Noble metals (Pt, Pd, Rh, Ru) as catalyst-electrodes have been mainly investigated to establish the mechanism [7], since they show weak adsorption and a low rate of nitrate reduction and a significant contribution of HER. In terms of efficiency and cost, Cu- and Co-based catalysts are of interest [21,25]. Copper, along with its alloys with Fe, Co, Ni, and noble metals, copper oxides, and Cu single-atom catalysts, has been reviewed [21]. Cu SAC achieved a maximum conversion of NH_3_.

The application of pure cobalt electrocatalysts encounters problems related to low conductivity and low stability in acidic media. The use of functionalized multi-walled carbon nanotubes (MWCNTs) as carriers for active cobalt catalysts demonstrated an FE of 84.72% in 0.1 M KOH with 0.1 M NO_3_^−^ at −0.16 V vs. RHE. Surface composition analysis by X-ray photoelectron spectroscopy (XPS) showed the presence of Co_3_O_4_ [26]. A comparison of 12 common transition metal oxide catalysts for NO_3_RR at a high cathodic current density was carried out [27]. The Co_3_O_4_ catalyst shows the highest FE of 85.15% at −0.25 V vs. RHE.

The study and application of Fe-containing catalysts for the electrochemical reduction of nitrate to ammonia has so far received insufficient attention compared to other base metals. Iron as a catalyst is employed in industry (Haber–Bosch process) and is present in various forms as a catalyst in the environment (nitrogenase enzyme) [28]. The disposal of such catalysts and release into the environment is not a factor that seriously limits the development and application of these electrocatalysts. The review [29] reported the use of pure iron, nanocomposites, bimetallic catalysts, and iron oxides in the NO_3_RR reaction. The FE was in the range of 74–98%. Pure iron has low stability in aqueous media due to corrosion. Nanocomposites and bimetallic catalysts, as well as the reasonable design of iron single-atom catalysts (Fe SAC) [30], can be a solution to this problem. For Fe SAC, the maximum FE was ~75% at −0.66 V with the highest NH_3_ production rate of 0.46 mmol/h/cm^2^ at −0.85 V. The rate of ammonia production on Fe SAC is not high enough, so the rational choice of a carbon substrate, an increase in the Fe SAC content on the substrate, and the introduction of components preventing the aggregation of Fe SAC particles is important. Iron is used in alloys or bimetallic catalysts to increase the efficiency of NO_3_RR.

According to [28], the proximity of the energies of the d-orbitals of Cu with the lowest unoccupied molecular π* orbitals of NO_3_^−^ facilitates electron transfer, which makes Cu an attractive electrode-catalyst. A dual-atom catalyst Fe/Cu due to the improved electron transfer on Cu demonstrates an FE of 92.51% for NRR under alkaline conditions.

In conclusion, the turn to non-platinum group metals, especially catalysts based on Fe, Co, Cu, and Ni, has opened more economical approaches to NO_3_RR since adsorption of *NO_3_ and its conversion to *NO_2_ occurs readily on the 3D-transition metal centers, and these metal centers play a crucial role mainly in the adsorption pathways at the O- and N-ends leading to the formation of NH_3_ [31].

The aim of this work was to obtain (one-step synthesis) and characterize a model bicatalyst for NO_3_RR, which is in demand for its composition and, first of all, will serve as a starting point for future work on NO_3_RR, NO_2_RR, and NRR. As shown above, Fe- and Fe/Co-containing electrocatalysts are not yet as well studied as, for example, systems containing Cu and Cu/Co, and even more so noble metals. Nanoparticles of Co, Fe, and bimetallic Fe/Co catalysts were obtained by the method of electrodeposition from aqueous solutions on a graphite substrate. Mono- and bicatalysts were tested in the reaction of electro-reduction of nitrate into ammonia and experimental data were obtained, which allowed us to suggest the nature of active metal nanoparticles (NPs).

## 2. Results and Discussion

### 2.1. Structure and Composition Characterization of Electrocatalysts

#### 2.1.1. SEM Analysis

**Figure 1:**
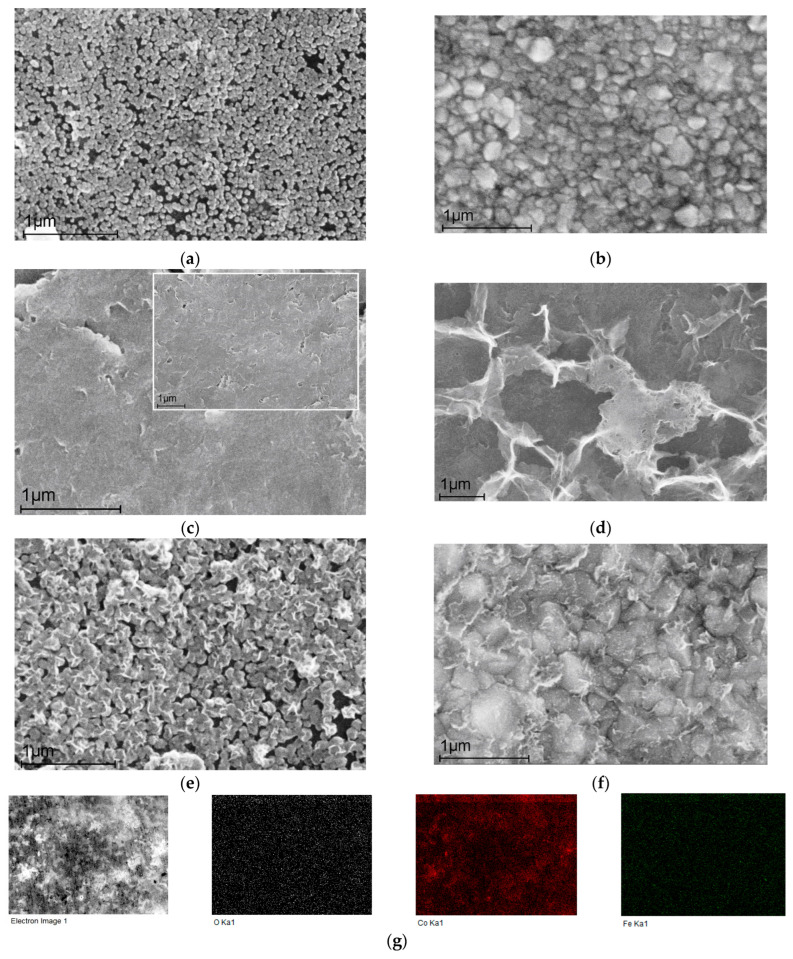
SEM images: (**a**) Co-NPs deposited for 5 min on a graphite substrate; (**b**) Co-NPs deposited for 30 min on a graphite substrate; (**c**) Fe(5min)/C; (**d**) Fe(30min)/C; (**e**) Fe(5min)@Co(5min)/C; (**f**) Fe(5min)@Co(30min)/C; (**g**) elemental mapping for Co, Fe, and O elements on a carbon substrate for Fe(5min)@Co(30min)/C.

Scanning electron microscopy images are presented in Figure 1. The electrochemically deposited cobalt nanoparticles (for 5 min, from 0.1 M FeSO_4_ aqueous solution (Section 3.2. Catalyst Preparation)) represent nanospheres with an average diameter of about 60 nm (Figure 1a). This fragmentary coating allows us to observe the graphite substrate (numerous black gaps between the nanoparticles), and, in this case, there is no complete isolation and therefore no absence of substrate influence. In contrast, dense surface coverage reliably isolating the carbon substrate with cobalt nanoparticles (deposition time 30 min) is shown in Figure 1b. The particles have lost their uniformity and are irregular spheres of variable diameters from 80 to 160 nm.

Figure 1c presents a surface image of a graphite substrate with electrodeposited (for 5 min) Fe-NPs, which have a dendrite morphology in contrast to the nanospheres of Co-NPs in Figure 1a,b. The inset demonstrates uniform surface coating. An even better dendrite shape of Fe-NPs is shown in Figure 1d for the deposition time (t_dep_) of 30 min.

Figure 1e,f present bimetallic Fe/Co nanocatalysts. Co-NPs were first deposited (t_dep_ = 5 min for Figure 1e and t_dep_ = 30 min for Figure 1f). The surface morphology of these nano-layers follows that of the samples presented in Figure 1a,b. Then, an independent layer of Fe-NPs (t_dep_ = 5 min for each sample) was deposited on the cobalt layer to obtain a bimetallic catalyst. Figure 1e,f clearly demonstrate both Co-NP nanospheres and Fe-NP dendrites. Further investigations demonstrate the obvious advantages of bi-catalysts containing Co-NPs and Fe-NPs on their surface, which exhibit a synergistic electrocatalytic effect expressed in high Faradaic efficiency values.

The elemental mapping images in Figure 1g also confirmed the generally uniform distribution of the elements Co, Fe, and O on the carbon substrate, indicating that the active centers may probably be present in the form of both iron and cobalt oxides.

#### 2.1.2. The Electrocatalysts Used in the Study

The investigated catalysts as well as some features of their preparation are briefly listed in Table 1. The preparation in detail is described in Section 3.2. Catalyst Preparation.

#### 2.1.3. X-ray Photoelectron Spectroscopy (XPS)

**Figure 2:**
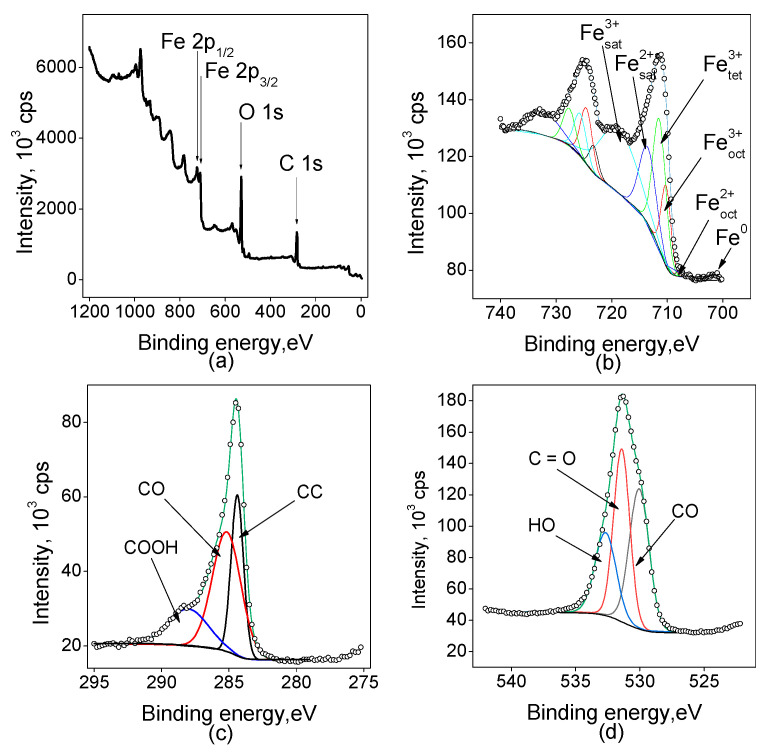
XP spectra for the sample Fe(5min)/C: (**a**) survey spectrum, and high-resolution spectra: (**b**) Fe 2p, (**c**) C 1s, (**d**) O 1s.

All the samples were investigated by the XPS technique to estimate the valence states of the elements on the surface of the electrodes. The spectra have a complex shape containing shake-up satellites (Figure 2).

Figure 2 shows the survey spectrum indicating the presence of C, O, and Fe, and high-resolution core-level photoelectron spectra in the regions of C 1s, O 1s, and Fe 2p for the sample Fe(5min)/C. The Fe 2p spectrum of the electrode demonstrates a typical spin-coupled doublet of Fe_3_O_4_ consisting of Fe 2p_3/2_ and Fe 2p_1/2_ components at binding energies of around 710 and 725 eV [32,33]. The deconvolution of the peaks shows the presence of both Fe^2+^ and Fe^3+^ components, the latter is present in both octahedral and tetrahedral coordination according to the literature [32]. The component of Fe^2+^ in the octahedral coordination is quite small, while the main peak area consists of about 1/3 Fe^3+^ in the octahedral coordination and 2/3 in the tetrahedral one. The O 1s region high-resolution spectrum also shows a multicomponent signal, which can be attributed to different oxygen-containing sites such as carbon–carbon bonds, carbon–oxygen, and so on. The ratios of different oxygen sites indicate that the surface is covered mainly with hydroxyl and carboxyl groups but C–O bonds are also present and contribute to about 25% of the spectral area. The C 1s spectrum analysis agrees with the interpretation of the oxygen 1s region: about half of the area is covered by a C-O component, while COOH groups contribute to about 20% of the area of the spectrum.

**Figure 3:**
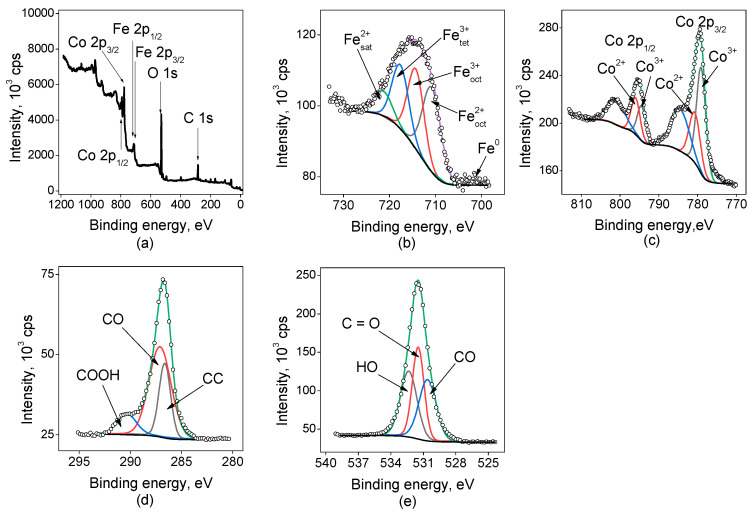
XP spectra for the sample Fe(5min)@Co(30min)/C: (**a**) survey spectrum, and high-resolution spectra: (**b**) Fe 2p_3/2_, (**c**) Co 2p, (**d**) C 1s, (**e**) O 1s.

Figure 3 shows the survey spectrum for the sample Fe(5min)@Co(30min)/C indicating the presence of C, O, Fe, and Co. In the case of this sample, the most abundant carbon species were C–O, they contribute to about 60% of the signal in the region of C 1s. The O 1s spectrum shows three bands corresponding to H–O, C–O, and C=O in near equal quantities. The Fe 2p spectrum is much wider than the previous ones and being deconvoluted the same way indicates the presence of a significant fraction of iron species in the state of Fe^2+^ in the octahedral coordination in an amount of about 40%, which is much more than the corresponding amount in the case of cobalt-free electrodes. But the reliability of such data is not clear. The fractions of Co^2+^ and Co^3+^ in the sample are nearly the same.

#### 2.1.4. X-ray Diffraction Analysis

**Figure 4:**
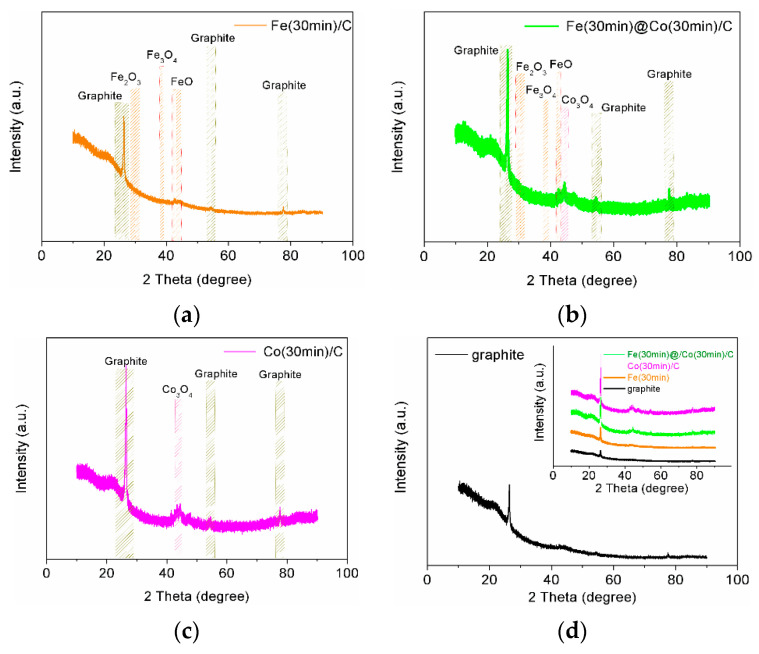
XRD pattern for electrocatalyst samples: (**a**) Fe(30min)C; (**b**) Fe(30min)@Co(30min)/C; (**c**) Co(30min)/C; (**d**) Graphite substrate and all samples in the insert.

Due to the extremely small number of deposited NPs, X-ray analysis showed weak reflexes for all catalyst samples, with the exception, of course, of the graphite substrate on which Fe- and Co-NP depositions took place. Additional deposition improved the appearance of reflexes, and the results are shown in Figure 4 (JCPDs 15-0806, JCPDs 06-0696, JCPDs 46-1312, JCPDs 21-0920). In general, we can say that these results do not contradict but rather confirm the more comprehensive complete data obtained using XPS analysis.

### 2.2. Linear Voltammetry Research

**Figure 5:**
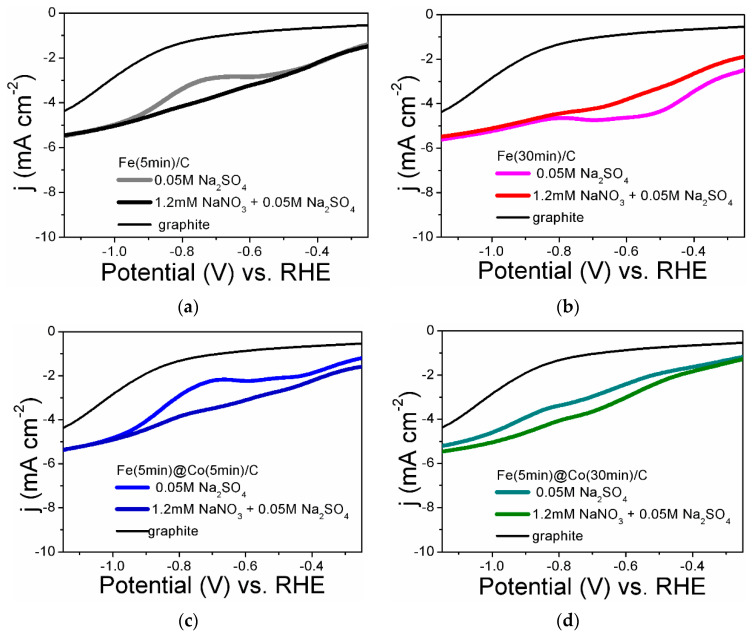
Linear voltammetric curves in Na_2_SO_4_ electrolyte containing and not containing nitrate ions at a potential scan rate of 50 mV s^−1^ for electrocatalyst samples: (**a**) Fe(5min)C; (**b**) Fe(30min)/C; (**c**) Fe(5min)@Co(5min)/C; (**d**) Fe(5min)@Co(30min)/C.

Figure 5 shows the LsVs obtained in the background electrolyte in the presence and absence of nitrate for all the electrocatalysts studied. For comparison, the LsVs obtained on a graphite substrate are shown in Figure 5a–d. As will be shown below, the substrate itself has a weak catalytic effect (FE from 0.8 to 5.4) in the investigated potential range (see Section 2.4. Analysis of Faradaic Efficiency). This effect can be eliminated by complete coating of the electrocatalyst surface with cobalt nanoparticles (Figure 1b,f). As can be seen from Figure 5, all catalysts show a rise in the current density in the presence of nitrate compared to the background electrolyte. The current rise is most evident in the studied potential region for Fe(5min)@Co(30min)/C and Fe(30min)/C. According to the literature, several reduction peaks on LsV correspond to the sequential reduction of nitrate to ammonia via intermediates [18,21]. For Fe(30min)/C, it can be assumed that the part of the current that contributes to side reactions (nitrogen release and HER) reduces, respectively, the ammonia yield in terms of the FE value. Thus, the Fe(5min)@Co(30min)/C bimetallic catalyst is more efficient.

### 2.3. Chronoamperometric Measurements and Nitrate Conversion

**Figure 6:**
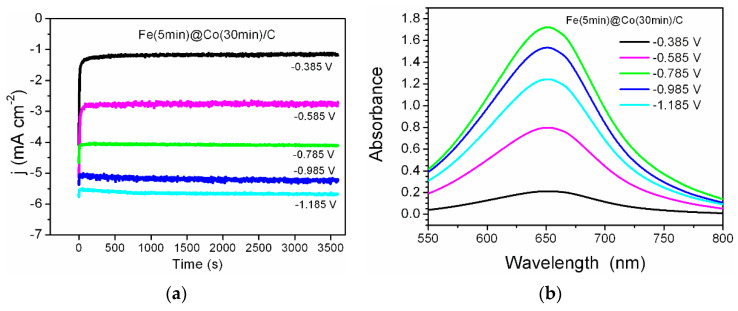
The process of ammonia synthesis by NO_3_RR for Fe(5min)@Co(30min)/C; at different potentials: (**a**) chronoamperometric curves in the 0.05 M Na_2_SO_4_ with 1.2 mM NaNO_3_ electrolyte; (**b**) UV–vis spectrum corresponding to the concentrations of the resulting product at λ = 652 nm.

Five potential values were chosen to perform NO_3_RR (t_reaction_ = 1 h) for each electrocatalyst from linear voltammetry. The selected potential values are in the interval from the double-layer (non-Faraday) region to the practical onset of hydrogen gas evolution. The NO_3_RR kinetics is close to zero at lower potential values, and, at higher ones, the side reaction of hydrogen evolution (HER) starts to significantly dominate. The chronoamperograms for the most efficient catalyst Fe(5min)@Co(30min)/C are shown in Figure 6a. The reaction time of NO_3_RR should be sufficient for the spectrophotometric detection of ammonia, and it was selected according to the literature data on modern recent works, for example, the research performed by Zhang et al. [26]. The UV–vis spectra corresponding to the concentration of ammonia synthesized in the NO_3_RR process are shown in Figure 6b. As can be clearly seen from Figure 6, the concentration maximum is observed at the middle values of the selected potential range (−0.785 V). This indicates a successful choice of the potential interval, where the optimum lies in the middle, with the reaction being too slow at the beginning of the range (−0.385 V) and HER starting to dominate at the end of the interval (−1.185 V).

### 2.4. Analysis of Faradaic Efficiency

**Figure 7:**
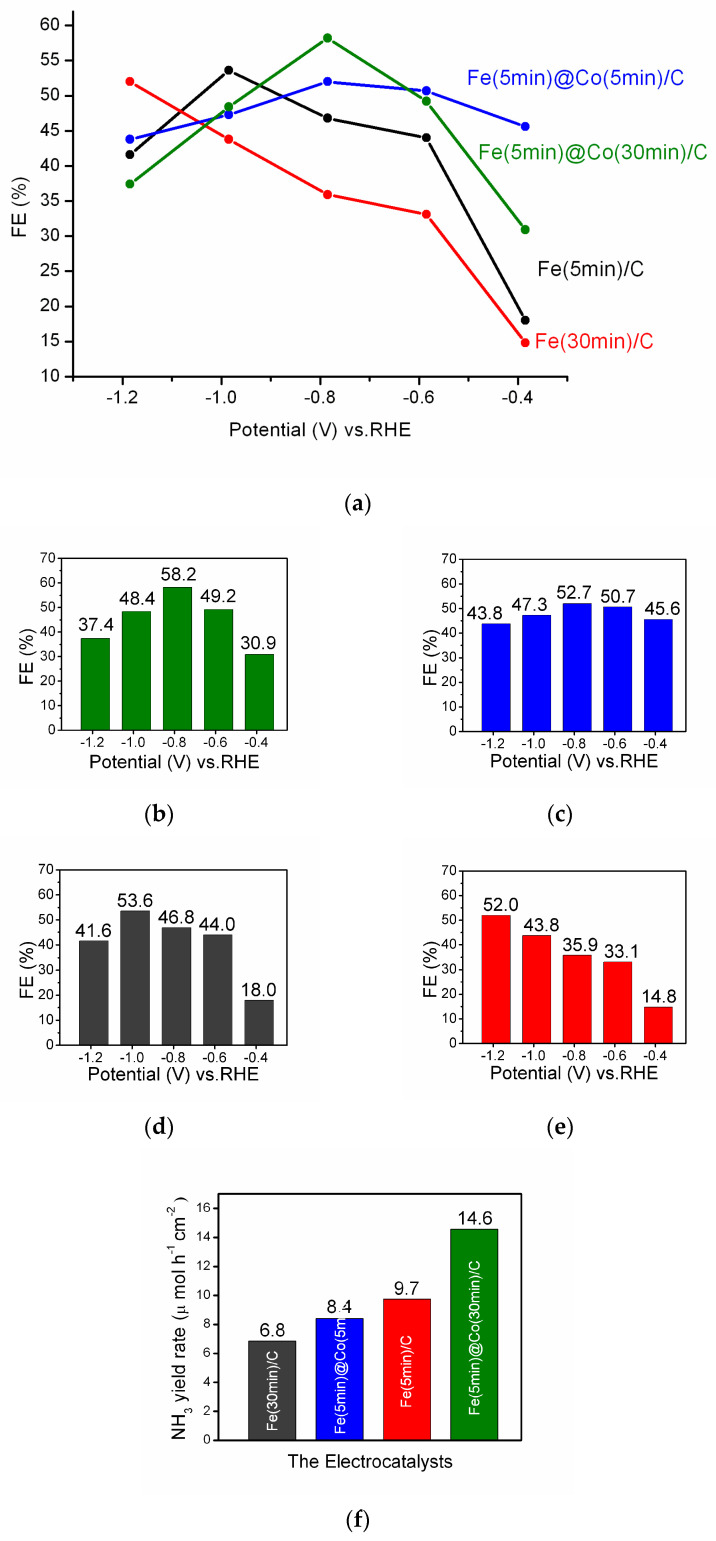
The values of FE and yield rate in NO_3_RR: (**a**) the resulting graph; and for single electrocatalysts: (**b**) Fe(5min)@Co(30min)/C, (**c**) Fe(5min)@Co(5min)/C, (**d**) Fe(5min)C, and (**e**) Fe(30min)/C. (**f**) NH_3_ yield rate of NO_3_RR at the potential −0.785 V.

Faradaic efficiency is an important characteristic for a description of the reaction selectivity, activity, and stability of the electrocatalyst [34,35]. The main results of the investigation are summarized in Figure 7a. For clarity and reader’s convenience, each FE result for each of the four electrocatalyst samples, Fe(5min)@Co(30min)/C (Figure 7b), Fe(5min)@Co(5min)/C (Figure 7c), Fe(5min)/C (Figure 7d), Fe(30min)/C (Figure 7e), is given for all the investigated potentials in separate figures. In general, the FE results, as a characterization of the electrocatalytic activity, showed good agreement with the LsV curves (Figure 5).

The Fe(30min)/C electrocatalyst (Figure 7e) shows almost a linear growth of FE, but exhibits the lowest efficiency in the potential range −0.4 to −1.0 V. Despite the considerably lower Fe content as a source of electrocatalytically active centers, the Fe(5min)/C catalyst has a higher FE value (Figure 7d) than Fe(30min)/C. This is probably due to the formation of more effective NO_3_RR catalytic centers than in a denser coating of Fe(30min)/C. The electrocatalyst Fe(5min)@Co(5min)/C (Figure 7c) containing thin layers of both Fe- and Co-nanoparticles shows a volcano-shaped plot for FE with a maximum at a potential of −0.785 V (Figure 7a). A clearly expressed volcano-like FE-E relationship (Figure 7a) and the highest FE result of 58.2% for E = −0.785 V (RHE) make the bimetallic catalyst Fe(5min)@Co(30min)/C the outstanding alternative in NO_3_RR compared to other catalysts investigated. The FE maximum for bimetallic catalysts is observed at a potential of −0.785 V, suggesting similarity in the mechanism and structure of the catalytic centers. The FE results are in correlation with the data presented in Figure 6. The catalyst Fe(5min)@Co(30min)/C exhibits the action of a true bimetallic catalyst, where the two components synergistically enhance the electrocatalytic properties of each other. The proposed mechanism of the bimetallic nanocatalyst in NO_3_RR based on the nature of the metal nanoparticles is discussed further below.

The ammonia yield rate for the four electrocatalysts is presented in Figure 7f. The bimetallic catalyst Fe(5min)@Co(30min)/C demonstrated the highest value of 14.6 µmol h^−1^cm^−2^ at the potential −0.785 V.

### 2.5. Determination of Electrochemically Active Surface Area (ECSA)

**Figure 8:**
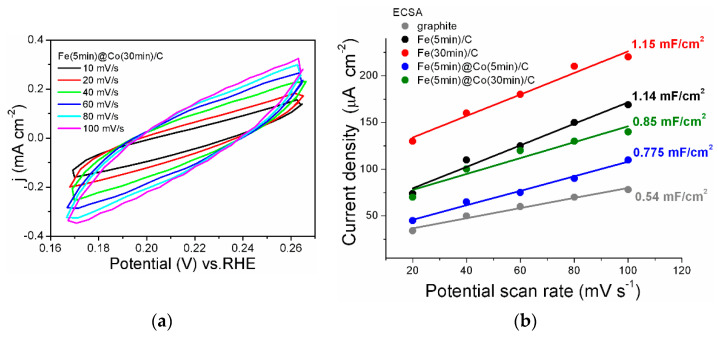
(**a**) Cyclic voltammograms for the Fe(5min)@Co(30min)/C electrocatalyst sample for a series at scan rates of 10, 20, 40, 60, 80, and 100 mV s^−1^ from 0.165 to 0.265 V (RHE). (**b**) The electrochemically active surface of the electrocatalyst samples presented as a double-layer capacity.

The electrochemical double-layer capacitance (Cdl) is an effective tool to estimate the electrochemically active surface area (ECSA) of catalysts, because ECSA is proportional to Cdl. For all the catalysts, cyclic voltammograms were obtained at scan rates of 10–100 mV s^−1^ in the non-Faradaic region, as presented in Figure 8a for the Fe(5min)@Co(30min)/C catalyst. A plot of the average current density vs. scan rate was built to obtain the Cdl values, as shown in Figure 8b. The values of the double-layer capacitance (Cdl) for all investigated samples including graphite are shown in Figure 8b. The Fe(30min)/C catalyst has a capacitance value of 1.15 mF cm^−2^, which is the best result for the whole series. This means that this sample has the most available catalytic centers, as does the other Fe(5min)/C sample (Cdl = 1.14 mF cm^−2^). It can be assumed that these catalytic centers are accessible not only for the reactants of the reaction under investigation, but also for other side processes (molecular nitrogen release, HER, corrosion [34]). Consequently, despite the lower value of Cdl = 0.85 mF/cm^2^ for the Fe(5min)@Co(30min)/C catalyst, its selectivity for the investigated reaction is significantly higher, as confirmed by the EF data discussed above.

### 2.6. Electrochemical Impedance Spectroscopy (EIS)

**Figure 9:**
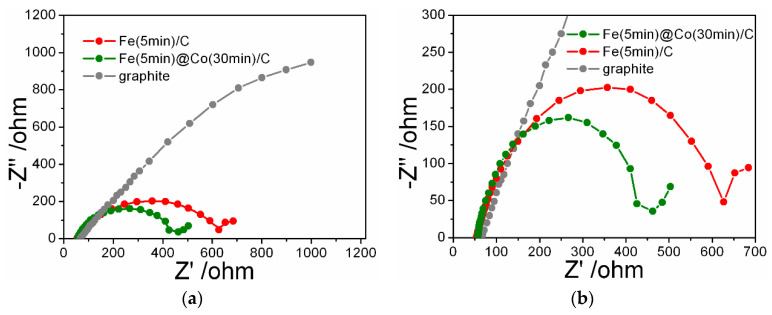
(**a**) Nyquist curves for electrocatalyst samples Fe(5min)@Co(30min)/C, Fe(5min)/C, and a graphite substrate in 1.2 mM NaNO_3_ with 0.05 M Na_2_SO_4_; (**b**) enlarged fragment.

The radius of Nyquist plots is related to the charge transfer resistance, a smaller radius indicates fast and efficient charge transfer during the NO_3_RR catalytic process (Figure 9a,b). The Nyquist plot for Fe(5min)@Co(30min)/C demonstrates that the combined presence of Co and Fe modifies the electrochemical properties of the electrodes. The catalyst containing only Fe has a higher resistance than the bimetallic catalyst, but at the same time, its resistance is much lower than that of graphite. The arc radius of Fe(5min)@Co(30min)/C is the smallest among the obtained catalysts, which indicates a low resistance to charge transfer and suggests that the addition of Co promotes charge transfer at the cathode and increases the reaction rate of conversion of nitrate to ammonia.

### 2.7. A Brief Summary of the Elucidation of the Proposed Mechanism of Electrocatalysis

According to [36,37,38,39,40,41], Fe-based electrocatalysts are able to accelerate all stages of NO_3_RR, and the reaction product can not only be ammonia but also nitrogen, which reduces the NH_3_ yield rate but is more acceptable from an environmental point of view for drinking water treatment [29]. It is believed that Fe^3+^, for the most part, catalyzes the early stages of NO_3_RR, and Fe^2+^ catalyzes the intermediate stages [36,37,38,39,40,41].

As seen in the XPS data (Section 2.1.3. X-ray Photoelectron Spectroscopy), Fe-NPs in Fe(5min)/C are present predominantly in the Fe^3+^ state, whereas they are found in the Fe^2+^ state (almost 40%) in Fe(5min)@Co(30min)/C. The addition of the Co-NP layer can be assumed to promote the adsorption of NO_3_^−^ and may catalyze the early steps of NO_3_RR and, in general, the process proceeds with a higher FE (Faradaic efficiency) and yield than without the Co-NP layer. This elucidation is, in principle, in good agreement with the EIS and ECSA data and can serve as a basis for understanding the role of the nature of NP metals.

## 3. Materials and Methods

### 3.1. Materials

The commercial cobalt (II) sulfate heptahydrate (CoSO_4_·7H_2_O, chemical purity), iron sulfate (II) heptahydrate (FeSO_4_∙7H_2_O, chemical purity), sodium nitrate (NaNO_3_, chemical purity), sodium sulfate (Na_2_SO_4_, chemical purity), boric acid (H_3_BO_3_, chemical purity), and graphite plates (mark V2L12) were used. All reagents were used without additional purification. Distilled water was employed for all experiments.

### 3.2. Catalyst Preparation

For the preparation of the electrocatalyst, graphite plates with dimensions of 50 × 7 × 1 mm were used as a support. Graphite plates were mechanically abraded and pre-cleaned, washed thoroughly with distilled water, and air-dried. Fe catalysts were prepared by electrodeposition on the graphite substrate from a 0.1 M FeSO_4_ aqueous solution under galvanostatic conditions at a current density of −1 mA cm^−2^ for 5 min or 30 min. The catalysts with Co-NPs were prepared by electrodeposition from an aqueous solution of 0.1 M CoSO_4_ with 1 M Na_2_SO_4_ as a background electrolyte and 0.5 M H_3_BO_3_ as a pH buffer [42]. Deposition of cobalt was carried out under potentiostatic conditions at E = –0.75 V (vs. Ag/AgCl) for 5 min and 30 min. A conventional cylindrical single-compartment (30 mL) electrochemical cell was used, where a platinum wire and a chlorosilver electrode (Ag/AgCl) were used as the counter electrode and reference electrode, respectively. The Autolab PGSTAT 302N potentiostat–galvanostat (Metrohm AG, Herisau, Switzerland) equipped with Nova 2.1.5 (The Netherlands–Switzerland) software was used for electrodeposition.

### 3.3. Electrochemical Measurements

Electrochemical measurements were carried out at room temperature using an Autolab PGSTAT 302N potentiostat (Metrohm AG, Herisau, Switzerland) with a three-electrode cell and an Ag/AgCl electrode as a reference electrode. Electrocatalysts on the graphite substrate were used as the working electrode, and platinum wire was used as the counter-electrode.

Linear voltammetry (LsV) over the range between (−0.25 V) and ≈(−1.185 V) vs. RHE at a potential scan rate of 50 mV s^−1^ was performed in a cathode–anode space-separated cell with a total volume of 60 mL.

Electrochemical reactions of NO_3_RR were carried out for 1 h in a cell with a separated cathode–anode space and a total volume of 60 mL. The electrolyte was a solution of 100 ppm (1.2 mmol/L) NaNO_3_ in 0.05 M Na_2_SO_4_, degassed by an Ar flow before the tests. All potential values were recalculated vs. the reversible hydrogen electrode (RHE) according to the formula E_RHE_ = E_applied Ag/AgCl_ + 0.202 + 0.059 × pH, unless otherwise noted.

Chronoamperometry tests were carried out in the potential range (−0.385 V) to (−1.185 V) vs. RHE for 1 h to determine the ammonia yield rates and Faradaic efficiencies.

### 3.4. Detection of Ammonia

**Figure 10:**
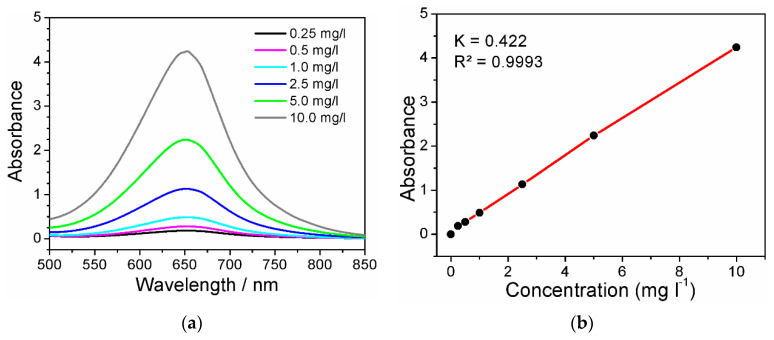
(**a**) UV–vis spectra, and (**b**) calibration line for testing NH_3_.

The detection of the ammonia content after NO_3_RR was carried out using the indophenol method, according to the methodology given elsewhere [43]. UV–vis absorption spectra (Figure 10a) were recorded using a Shimadzu 3600 Plus spectrophotometer (Shimadzu, Japan) in a standard 1 cm quartz cuvette. Two milliliters of 5 wt% sodium salicylate in 1.0 M NaOH was added to 2 mL of the tested solution, then 1 mL of 0.05 M NaClO and Na_2_[Fe(NO)(CN)_5_] (0.2 mL, 1 wt%) was added. The solutions were kept at 40 °C for 1 h. The absorption maximum is observed at λ = 652 nm, for which a calibration graph was plotted. The resulting calibration graph is described by the equation (y = 0.4217x + 0.0641; R^2^ = 0.9993) and shows a good linear relationship between the absorbance value and NH_3_ concentration in the range from 0.25 mg/mL to 10 mg/L (Figure 10b).

The Faradaic efficiency was determined by the formula:

(1) $$FE({NH}_{3})=\frac{8\times F\times n({NH}_{3})}{Q},$$

where *n*(*NH*_3_) denotes the amount (mol) of NH_3_; *F* is the Faradaic constant (96,485 C mol^−1^); *Q* is the total charge passed through the electrode, 8 is the number of electron (*n*) transfers required to form 1 mol of ammonia.

The ammonia *yield* (*NH*_3_) rate (yield) was defined as:

(2) $$yield({NH}_{3})=\frac{C{\left({NH}_{3}\right)}_{\;}\times V}{17\times t\times S},$$

where *C*(*NH*_3_) denotes the mass concentration (μg mL^−1^) of NH_3_ calculated from the UV–vis spectra, *t* is the electrolysis time; *S* is the geometric area of the working electrode (1 cm^2^); *V* is the volume of the electrolyte.

### 3.5. ECSA Evaluation

The *ECSA* value was calculated from the value of the double-layer electrochemical capacitance (*C_dl_*) obtained by measuring CV (cyclic voltammogram) in the double-layer potential range, i.e., the non-Faradaic area. All catalysts were scanned in the potential range from 0.165 V to 0.265 V vs. RHE in NaNO_3_ (1.2 mM) in 0.05 M Na_2_SO_4_ at different scan rates (10 to 100 mV s^−1^). The values of the current density at 0.215 V vs. RHE at different scan rates were calculated, and the curves of the dependence of scan rates for each catalyst were plotted. The dependences of the current densities on scan rates were obtained, and the *C_dl_* values were obtained accordingly. *ECSA* was calculated as

(3) $$ECSA=\frac{C_{dl}}{C_{s}}$$

where *Cs* (=0.4 F∙m^−2^) is the total specific capacitance for an atomically smooth planar surface under homogeneous electrolytic conditions.

### 3.6. Impedance Response Testing

Impedance spectra were measured in a three-electrode undivided cell (60 mL) at room temperature in a solution 1.2 mM of NaNO_3_ in 0.05 M Na_2_SO_4_. An Ag/AgCl reference electrode was used. The auxiliary electrode was a platinum wire. Measurements were carried out with a P-40X potentiostat with an electrochemical impedance measurement module FRA-24M (Electrochemical Instruments, Moscow, Russia) in the frequency range from 50 kHz to 0.01 Hz at an AC voltage amplitude of 20 mV. The time of immersion of the sample corresponded to the time of impedance measurement without preliminary exposure in the medium.

### 3.7. Material Characterization

Scanning electron microscopy (SEM) analysis was performed with a LEO EVO 50 xvp electron microscope (Carl Zeiss, Jena, Germany) equipped with an EDX (energy dispersive X-ray spectroscopy) analyzer (Zeiss AG, Jena, Germany).

The powder X-ray diffractograms (XRD) were obtained with an STOE STADI P diffractometer (STOE & Cie GmbH: Darmstadt, Germany) equipped with a Ge-monochromator, CuKα1 emission, λ = 1.54056 Ǻ, linear PSD in the transmittance geometry. The samples were examined in the region 2θ = 10–90° with a scanning step of 0.01° and an exposure time of 15 s per point. The samples were identified by comparing theoretical and experimentally obtained X-ray diffraction patterns using WinXPOW version 2.24.

XPS measurements were performed using a PREVAC EA15 spectrometer (PREVAC, Rogów, Poland). In the current work, AlKα radiation (hν = 1486.6 eV, 150 W) was used as a primary radiation source. The pressure in the analytical chamber did not exceed 5 × 10^−9^ mbar during spectra acquisition. The binding energy scale was pre-calibrated using the positions of Ag 3d5/2 (368.3 eV) and Au 4f7/2 (84.0 eV) from silver and gold foils, respectively. The powdered samples were supported onto a double-sided conducting scotch tape. To take into account the effect of surface charging, the C1s line at (Eb = 284.8 eV) from the carbon contamination was used as an internal standard.

## 4. Conclusions

Green and sustainable electrocatalytic conversion reactions of ammonia production are currently in high demand in order to replace the eco-unfriendly Haber–Bosch process. The model catalysts for the nitrate reduction reaction were synthesized by electrodeposition of metal nanoparticles Co, Fe, and bimetallic Fe/Co catalyst from aqueous solutions onto a graphite substrate. The main results of the completed study are as follows:

- The surface morphology and NP size, defining the further efficiency of electrocatalysts in NO_3_RR, were determined by scanning electron microscopy. XPS and XRD revealed the state and composition of catalytic nanoparticles.
- According to the results of linear voltammetric studies, five potential values were selected at which NO_3_RR was performed for 1 h for each sample of the electrocatalyst.
- A clearly expressed volcano-like FE-E relationship (Figure 7a) and the highest FE result of 58.2% for E = −0.785 V (RHE) and ammonia yield rate of 14.6 μmol h^−1^ cm^−2^ highlight the Fe(5min)@Co(30min)/C bimetallic catalyst in NO_3_RR compared to other investigated catalysts.
- The ECSA method showed that despite the lower value of Cdl = 0.85 mF/cm^2^ for the Fe(5min)@Co(30min)/C catalyst, its selectivity for the investigated reaction is significantly higher, as confirmed by the EF data discussed above. It was found by the EIS method that the addition of a Co-nanolayer promotes charge transfer at the cathode and increases the reaction rate of conversion of nitrate to ammonia.
- The nature and morphology of Fe- and Co-nanoparticles suggest a joint catalysis to accelerate the early and intermediate stages of NO_3_RR.

This work can serve primarily as a starting point for future investigations on electrocatalytic conversion reactions of ammonia production using model catalysts of the proposed type.

## Figures and Tables

**Table 1:** The Samples of electrocatalysts and features of their preparation.

Designation	General Brief Description
C	Graphite is the initial substrate
Fe(30min)/C	Fe-NPs (deposited at 30 min) on the substrate C
Fe(5min)/C	Fe-NPs (deposited at 5 min) on the substrate C
Fe(5min)@Co(5min)/C	Fe-NPs (deposited at 5 min) on the Co-NP layer (deposited at 5 min) on the substrate C
Fe(5min)@Co(30min)/C	Fe-NPs (deposited at 5 min) on the Co-NP layer (deposited at 30 min) on the substrate C

## Data Availability

Data is contained within the article.
